# Gene Regulation by the LiaSR Two-Component System in *Streptococcus mutans*

**DOI:** 10.1371/journal.pone.0128083

**Published:** 2015-05-28

**Authors:** Manoharan Shankar, Saswat S. Mohapatra, Saswati Biswas, Indranil Biswas

**Affiliations:** Department of Microbiology, Molecular Genetics and Immunology, University of Kansas Medical Center, Kansas City, Kansas, United States of America; University of Rochester Medical Center, UNITED STATES

## Abstract

The LiaSR two-component signal transduction system regulates cellular responses to several environmental stresses, including those that induce cell envelope damages. Downstream regulons of the LiaSR system have been implicated in tolerance to acid, antibiotics and detergents. In the dental pathogen *Streptococcus mutans*, the LiaSR system is necessary for tolerance against acid, antibiotics, and cell wall damaging stresses during growth in the oral cavity. To understand the molecular mechanisms by which LiaSR regulates gene expression, we created a mutant LiaR in which the conserved aspartic acid residue (the phosphorylation site), was changed to alanine residue (D58A). As expected, the LiaR-D58A variant was unable to acquire the phosphate group and bind to target promoters. We also noted that the predicted LiaR-binding motif upstream of the *lia* operon does not appear to be well conserved. Consistent with this observation, we found that LiaR was unable to bind to the promoter region of *lia*; however, we showed that LiaR was able to bind to the promoters of SMU.753, SMU.2084 and SMU.1727. Based on sequence analysis and DNA binding studies we proposed a new 25-bp conserved motif essential for LiaR binding. Introducing alterations at fully conserved positions in the 25-bp motif affected LiaR binding, and the binding was dependent on the combination of positions that were altered. By scanning the *S*. *mutans* genome for the occurrence of the newly defined LiaR binding motif, we identified the promoter of *hrcA* (encoding a key regulator of the heat shock response) that contains a LiaR binding motif, and we showed that *hrcA* is negatively regulated by the LiaSR system. Taken together our results suggest a putative role of the LiaSR system in heat shock responses of *S*. *mutans*.

## Introduction

Two-component systems (TCS) in pathogenic bacteria are key factors that dictate survival in hostile niches, as they are necessary to sense and respond to environmental stresses. These systems are also known to regulate the expression of multiple virulence factors in a variety of human pathogens. The regulatory cascade effected by these systems typically involves two proteins: a sensor kinase, that autophosphorylates in response to the stress and transfers phosphate to a response regulator, which then dimerizes and binds to specific sequences in the promoter of its target genes and modulates their expression. Although autophosphorylation by the sensor kinase and phosphotransfer to the cognate response regulator are considered key steps in the regulatory cascade, response regulators are also known to acquire the phosphate group from other non-cognate sensor kinases and from cellular small molecular phosphodonors such as acetyl phosphate [1]. TCS in pathogens regulate chemotaxis; biofilm formation; resistance to environmental stresses (pH, temperature, salinity, osmolarity, antibiotics, and others); biotic stresses (host antimicrobials and other competing microbes) and therefore are critical for persistence in the niche [2]. Thus, drugs targeting these systems are becoming attractive options in the efforts to curb virulence and persistence [3, 4].

The LiaSR constitute a TCS that is widely found in Gram-positive bacteria with low G+C content and is known to regulate multiple targets that determine virulence, stress tolerance and persistence in these bacteria [5–10]. The LiaSR system has been most studied in *Bacillus subtilis* where it is encoded as a part of the *liaIHGFSR* operon. Deletion of several genes in this operon led to increased sensitivity to cell wall targeting antibiotics [9–12]. Of these, *liaF* has been consistently found upstream of *liaSR* suggesting that it might play a role in functioning of the LiaSR pathway. Subsequently, deletion of *liaF* was shown to deregulate expression from the *liaIHGFSR* promoter suggesting that LiaF could negatively autoregulate the operon [9, 11]. A striking characteristic of the LiaSR system is that its expression is induced upon exposure to antibiotics that target the cell envelope by interfering with the lipid II cycle of cell wall biogenesis (bacitracin, vancomycin, and others) [5, 6, 10, 13]. Orthologs of the *liaSR* genes have been found in several pathogenic bacteria and shown to be involved in sensing cell-wall, antibiotic, acid, and detergent stresses. *Staphylococcus aureus* for example, harbors the VraSR system and mutations in this TCS have been shown to be involved in increasing resistance to antibiotics [14–16]. Orthologs have also been detected and characterized in the food borne pathogens *Listeria monocytogenes*; *Enterococcus* sp., where multi-drug resistance is evolving rapidly; and in streptococci, where the role of LiaSR has been implicated in acid, detergent and antibiotic stress response [6, 13, 17, 18].

In *S*. *mutans* UA159, LiaS and LiaR are expressed from a three-gene operon (*liaFSR*: SMU.485, SMU.486 and SMU.487) along with the LiaF. LiaF functions as an inhibitor of liaFSR expression both in *S*. *mutans* [5] and in *B*. *subtilis* [9], where it is believed to affect the functioning of LiaS [19]. Reverse transcriptase PCR and Northern blotting have indicated earlier that the *liaFSR* operon is transcriptionally fused to downstream genes SMU.488 and SMU.489 and produces a pentacistronic transcript [5]. Earlier work from our lab suggests that inactivation of *liaS* provided the mutant strain with a growth advantage in the presence of antibiotics and inhibitors of DNA replication as compared to the wild type [20]. LiaS has also been shown to negatively regulate the expression of a glucan binding protein (*gbpC*), which is critical for adhesion to surfaces; and to positively regulate production of mutacin IV [21]. Inactivation of *liaR* however, did not affect the expression of any of the virulence factors, suggesting that either LiaS could involve in cross-talk with other TCS or that LiaS inactivates LiaR function [21]. Global expression profiling of a *liaR* deletion strain of *S*.*mutans* UA159 under biofilm formation conditions has revealed a host of 174 genes possibly regulated by LiaR either directly or indirectly [22]. Only a few regulons have been predicted to be directly regulated by LiaR including SMU.485 (LiaF), SMU.753 (a PspC domain containing protein), SMU.1727 (Oxa2 class transcriptional regulator) and SMU.2084 (SpxB regulatory protein involved in cell wall homeostasis) [5, 6].

Since most of the fundamental functional aspects of the LiaSR TCS in *S*. *mutans* were extrapolated from homologs in other bacteria, we revisited the function of this TCS by attempting to clarify the existing ambiguities. Though several regulons of LiaR have been predicted and identified in *S*. *mutans*, it is still unclear whether these regulons are directly under the control of the LiaR or are indirectly regulated by one of the transcriptional regulators under the control of the LiaSR TCS [5, 22]. This complicates the identification of a consensus LiaR binding motif. LiaR binding sites have been predicted in *L*. *Lactis* (CesR) and *S*. *pneumoniae* by independent groups largely based on the presumption that LiaR binds to the *liaFSR* promoter (P_*SMU*.*485*_) and regulates the expression levels of its own operon (*liaFSR*) [5, 6]. These predicted sites, when compared to the LiaR-binding site in P_*SMU*.*485*_ differs by a few key residues that were fully conserved in other promoters. In this study, by segregating the direct regulons of LiaSR in *S*. *mutans* we have newly generated a LiaR-binding consensus and redefined regulation by this TCS.

## Material and Methods

### Bacterial strains and growth conditions

*S*. *mutans* strains were grown in Todd-Hewitt medium (BBL, BD) supplemented with 0.2% yeast extract (THY). THY broth was supplemented with 300μg/ml kanamycin (Km) or 5μg/ml erythromycin when necessary. *E*. *coli* strains were routinely grown in Luria-Bertani medium supplemented with 50μg/ml Km, 100μg/ml Ampicillin (Ap) or 300μg/ml Em as required.

### Construction of a *liaR* clean deletion mutant

To construct IBSA13 (Δ*liaR*) strain, we first amplified the entire *liaFSR* region (~3.2kb) with primers SMU484L486F2 and BamSMU487R2 (for all primers, see Table 1) and the resultant fragment was cloned into pGemTEZ vector (Promega, USA) to create pIBA3. Plasmid pIBA3 was restricted with *Nhe*I and *Bbs*I and a loxP-Km cassette ([23]) was inserted after blunting to generate pIBA9.2. S. muatns UA159 was transformed with pIBA9.2 after linearizing with *Not*I and Km resistant transformants were selected and verified for the insertion of the Km cassette by PCR. Subsequently, the Km cassette was excised out with the help of pCrePA as described before [23]. The resultant clean *liaR* deletion strain was verified by PCR followed by sequencing the entire *lia* operon.

**Table 1:** List of oligonucleotides used in this study.

Primer Name	Sequence (5’-3’)	Target / Purpose
EcoRSMU487F1	GCGGAATTCATGCTGATGAGTAAAACAAAAGTTATACTGG	*liaR* expression
BamSMU487R2	CGCGGATCCAACTCTGTTGGTTTTGAAACAACAGC	*liaR* expression
LiaRD58AF	GACGTTGTTGTCATGGCCTTGGTTATGCCAGAA	D58E mutant creation
LiaRD58AR	TTCTGGCATAACCAAGGCCATGACAACAACGTC	D58E mutant creation
SMU486ECOEXF1	GCGGAATTCCTCAACCAAAATTTAAAACGCATTCTGC	*liaS* expression
SMU486HINDEXR1	CGCGAAGCTTTTACTCATCAGCATCCCCTTTCATTATTGG	*liaS* expression
4QPsmu.485F	CATGTCAATTCAAGTTATGTTTATG	P_*SMU*.*485*_ amplification
4QPsmu.485R	AGCTTAGTTATCATACATCAATCTTAT	P_*SMU*.*485*_ amplification
4QPsmu.753F	ATTACCAATGTGAATCGTGTGG	P_*SMU*.*753*_ amplification
4QPsmu.753R	ACAAACAACTCTCCCTTTTTTA	P_*SMU*.*753*_ amplification
4QPsmu.1727F	GTTTATTCAAGAAATTCGCAAAG	P_*SMU*.*1727*_ amplification
4QPsmu.1727R	GATAATCTTCCTTTGATTTTAATAAT	P_*SMU*.*1727*_ amplification
4QPsmu.2084F	TTAATTGAGGAAGATAATACTGAAG	P_*SMU*.*2084*_ amplification
4QPsmu.2084R	GTTTAAATCTCTCTTTCCTTACAG	P_*SMU*.*2084*_ amplification
4QLiaIntF	CTTGTTTGAAGAGATAACTTTTTTG	Alt. P_*SMU*.*485*_ amplification
4QLiaIntR	CATAAGGCTTCCTTCAGTTCTAC	Alt. P_*SMU*.*485*_ amplification
4QPhrcAF	ACAGCAATAAAACTAAGTCTTAAGTC	P_*hrcA*_ amplification
4QPhrcAR	CCTAAGTCCACCTCCCATC	P_*hrcA*_ amplification
4QRThrcAF	GTGGAGCCTAAAAAGCAACGTT	*hrcA* (qPCR)
4QRThrcAR	GAATGGCAAATTGCACCGTTA	*hrcA* (qPCR)
4QRTrpoBF	AGCCATCTCACCCTTTTCCAT	*rpoB* (qPCR)
4QRTrpoBR	CCGTCGTTCTAACTCTGGAACTG	*rpoB* (qPCR)

### LiaR protein expression and purification

The LiaR encoding ORF was amplified from the *S*. *mutans* UA159 chromosome by PCR using primers EcoRSMU487F1 and BamSMU487R2, digested with *Bam*HI and *Eco*RI and ligated to the tetracycline-inducible protein expression vector pASK-IBA43+ (IBA, Germany) digested with the same enzymes to generate pIBE11. *E*. *coli* BL21 cells containing the resulting construct pIBE11 were used to express the recombinant protein fused to an N-terminal histidine tag. Protein expression was induced in cultures at an OD_600_ of ~0.6 using anhydrotetracycline (0.2μg/ml) for 4hrs at 30°C. The recombinant protein was purified by affinity chromatography using nickel-NTA resin following standard protocol. Plasmid pIBE11 was used as template in a long-range PCR reaction with mutagenic primers (LiaRD58AF and LiaRD58AR) to amplify the entire plasmid. These primers introduced a C→A mutation at position 173 in the *liaR* ORF, thereby causing a D58A replacement in the protein sequence. After sequencing both strands to verify the mutation, this construct, pIBE29, was used for the recombinant expression of LiaR-D58A protein. A fragment containing *lia*S was amplified from *S*. *mutans* UA159 chromosome using primers SMU486ECOEXF1 and SMU486HINDEXR1, and was digested with *Hind*III and *Eco*RI and ligated to the IPTG-inducible protein expression vector pMalC4 (NEB, USA) to yield pIBA13. The recombinant protein, fused to a maltose binding protein (MBP) tag was purified by affinity chromatography on amylose resin.

### Phosphorylation and phosphotransfer assays

They were carried out as described earlier [24] with some modifications. To demonstrate autophosphorylation, purified LiaS at a final concentration of ~1μM was incubated in phosphorylation buffer 1 containing 20mM Tris-Cl, 50mM KCl, 10mM MnCl_2_, and 1mM DTT in the presence of 10μCi γ^32^P-dATP for varying times. Phosphotransfer from LiaS to LiaR was demonstrated by incubating a mixture of 1μM-phosphorylated LiaS and 2μM LiaR in phosphorylation buffer 1 for varying times. The reaction products were resolved on SDS-PAGE (10%) gels, which were dried, exposed to phosphor screens and imaged on a Typhoon FLA 9500 biomolecular imager. To assess LiaR phosphorylation by small phosphodonors, ~5μg of purified His-LiaR or His-LiaR-D58A were incubated in phosphorylation buffer 2 containing 50mM Tris-Cl, 50mM KCl, 20mM MnCl_2_, 1mM DTT at pH 7.5, with or without 50mM acetyl phosphate (Ac-Po4) or 20mM phosphoamidate (PA, [25, 26]) for 1 h at 37°C and then resolved by Phos-Tag-SDS PAGE [27].

### Electrophoretic mobility shift assays (EMSA)

EMSA were carried out as described previously [28] with minor modifications. EMSA reactions were conducted in binding buffer containing 20mM Tris-Cl, 100mM NaCl, 0.01mM DTT, 5% glycerol (vol/vol), 1mM EDTA, 0.01mg/ml BSA, 5mM MgCl2, and 10μg/ml poly (dI-dC) at pH 7.5. ~200bp DNA fragments suspected to contain LiaR binding sites were amplified by PCR using specific primers (Table 1), gel purified, end labelled with γ^32^P-dATP and used in the assay. ~0.5pmol of purified DNA was incubated in binding buffer with 0, 5, 10 and 15pmol of purified His-LiaR or His-LiaR-D58A for 30min. Cold competition assay was performed using non-radiolabelled P_*SMU*.*2084*_ probe as the cold competitor in an EMSA reaction with radiolabelled P_*SMU*.*2084*_ probe. Oligo pairs of the predicted LiaR binding motif with or without alterations were annealed in annealing buffer containing 10mM Tris-Cl, 50mM NaCl and 1mM EDTA at pH 7.5 by heating equimolar mixtures at 95°C and allowing them to cool down gradually over a period of 45min. Annealed oligos were end labelled with γ^32^P-dATP and used in the assay. ~1pmol of double stranded annealed oligos was incubated in the presence of 0, 15, 20 and 30pmol of purified His-LiaR in binding buffer for 30min. Binding reaction mixtures with ~200bp DNA fragments and ~25bp annealed primers were resolved on chilled 5.5% and 7.5% (wt/vol) polyacrylamide gels respectively using 0.5x TBE buffer containing 5% (vol/vol) glycerol.

### Prediction of the LiaR binding motif and potential LiaR regulons

Analysis was done using programs in the Multiple EM by Motif Elicitation (MEME) suite (http://meme.nbcr.net/meme) [29]. To identify the LiaR-binding motif, ~200bp regions upstream of known LiaR regulons SMU.753, SMU.2084 and SMU.1727 were submitted to the MEME tool. A maximum of 10 motifs were to be detected in the size range of 20 to 50bp at an occurrence rate of 0 or 1 per sequence. The motif detected in all three submitted sequences with the lowest E-value was chosen as input for a FIMO (Find individual motif occurrences) search of the *S*. *mutans* UA159 genome (Genbank: NC_004350.2). Motif occurrences with a P-value lower than 1e^-5^ were inspected manually to determine their positioning upstream of the regulons in order to qualify as a potential regulon.

### Biolayer interferometry (BLI)

BLI, a label-free, biosensor-based method, where one of the interacting partners is immobilized on the biosensor (ligand) and the analyte is in solution [30] was used to measure the binding kinetics of the LiaR-DNA complex in real time. BLI was performed using annealed 25bp test primers that were previously biotinylated on one strand at the 3’ end. Annealed primers, prepared in binding buffer, were quantified and loaded on hydrated (10min in binding buffer) streptavidin biosensors for 5 min. A baseline step was included between the loading and association steps to remove any unbound / excess primers from the biosensor tips by exposing the tip to binding buffer for 2min. Association was performed for 5min by exposing the biosensor tip to LiaR solutions of varying concentrations prepared in binding buffer. Dissociation of the DNA-LiaR complex was assessed by exposing the complex bound to the biosensor tip to binding buffer for 5min. Association and dissociation data was collected in real-time and used to compute the maximum LiaR binding ability (R_max_) and equilibrium LiaR binding ability (R_eq_) of the oligos to be tested. R_max_ and R_eq_ values were calculated by the BLITZ Pro software (v1.1).

### Quantitative PCR

Primer pairs producing ~120bp products were designed for *hrcA* and the endogenous control *rpoB* using Primer Express v3.0 (Table 1). The qPCR assays were done as described earlier with some modifications [31]. *S*. *mutans* UA159 and its isogenic *liaR* deletion mutant IBSA13 were grown in THY broth for 16 h at 37°C. Fresh THY broth was seeded with 1% inoculum from the starter culture and allowed to reach an OD_600_ of ~0.85 before being harvested for RNA extraction using the Qiagen RNAeasy mini kit. DNA contamination, if any was removed by RNase-free DNase (Qiagen) treatment following which, each purified RNA sample was quantified. First strand synthesis was carried out from 2μg of RNA template using the RevertAid first strand cDNA synthesis kit. qPCR was set up including appropriate negative controls, using the DyNAmo flash SYBR green qPCR kit according to manufacturer’s instructions on an Applied Biosystems 7500 real time PCR system, supplying ~2.5ng of cDNA per μl of reaction. The wild-type gene expression level was used as the calibrator to detect the relative change in gene expression in the *liaR* mutant.

## Results

### LiaS autophosphorylates and transfers phosphate to LiaR

**Fig 1:**
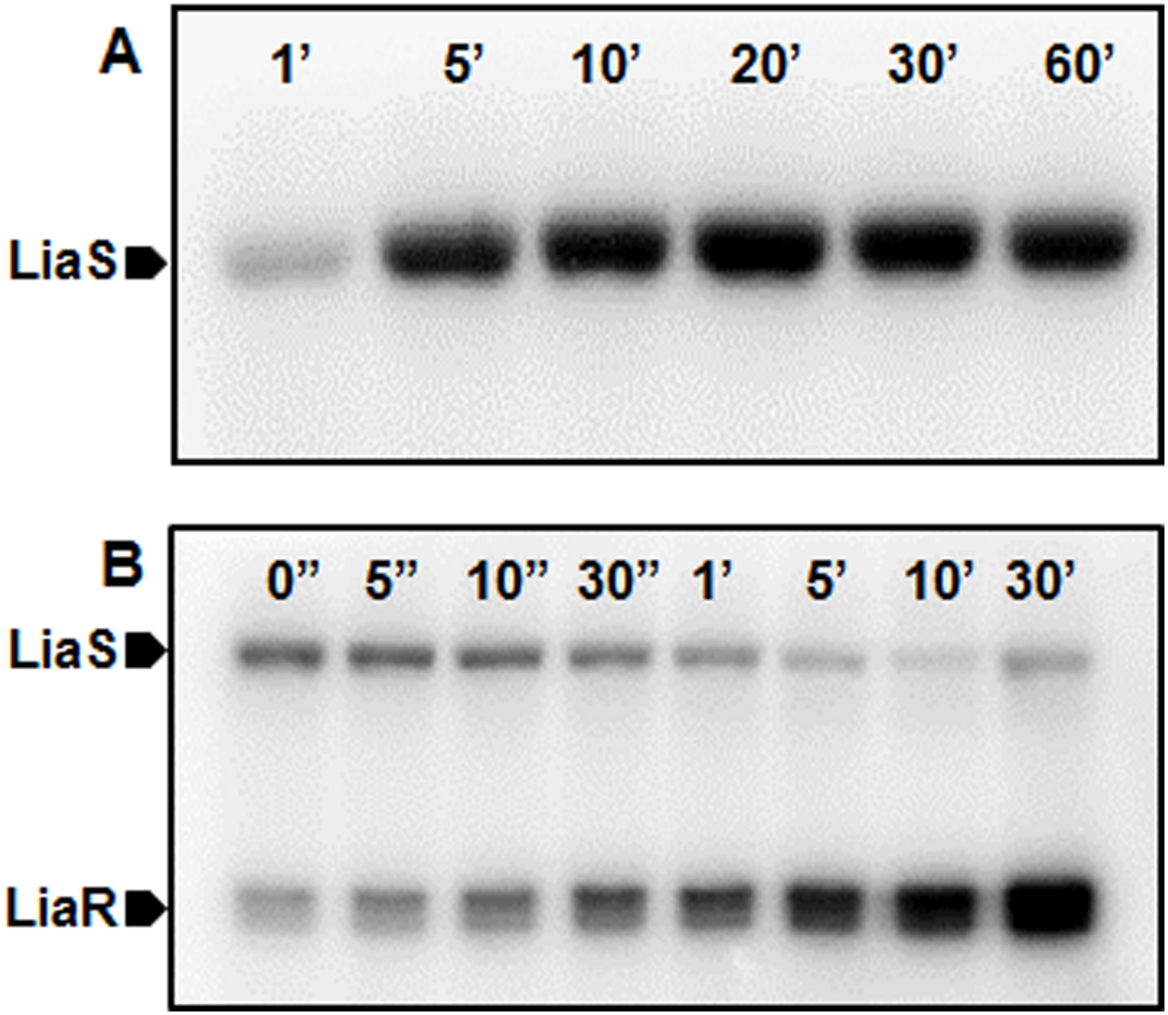
LiaS autophosphorylates and transfers phosphate to LiaR. (A) MBP-tagged LiaS was incubated in phosphorylation buffer 1 (20 mM Tris-Cl, 50 mM KCl, 10 mM MnCl_2_, 1 mM DTT and 10 μCi γ^32^P-dATP at pH 7.4) for indicated times. (B) MBP-tagged LiaS, pretreated with γ^32^P for 10 min was incubated with His-tagged LiaR (molar ratio 1:2) in phosphorylation buffer 1 for indicated times. Both reaction products were resolved on 10% SDS-PAGE gels.

The classic model of a TCS involves gene regulation by a response regulator protein after being phosphorylated by its cognate histidine kinase. To understand if the LiaSR TCS in *S*. *mutans* functions similarly, we performed *in-vitro* auto-phosphorylation and phosphotransfer assays for the LiaS and its cognate response regulator LiaR. Autophosphorylation by the LiaS was detectable within a minute of γ^32^P-ATP exposure and remained stable throughout the observed 1 h period (Fig 1A). Co-incubation of pre-phosphorylated LiaS with LiaR led to a progressive loss of LiaS phosphorylation signal, but was accompanied by a simultaneous increase in the phosphorylation of LiaR over time, suggesting phospotransfer from LiaS to LiaR. Phosphotransfer to LiaR was detectable almost immediately after mixing LiaS and LiaR. LiaR that was phosphorylated by LiaS remained detectable over the entire 30min period of observation indicating stability of the transferred phosphate group (Fig 1B). Further analysis of the LiaR protein and its orthologs identified a conserved aspartic acid residue at position 58 (D58) as the potential site of phosphorylation. This led us to probe the essentiality of this residue in phosphorylation and promoter binding by LiaR.

### Aspartate at position 58 (D58) is essential for LiaR phosphorylation and DNA binding

**Fig 2:**
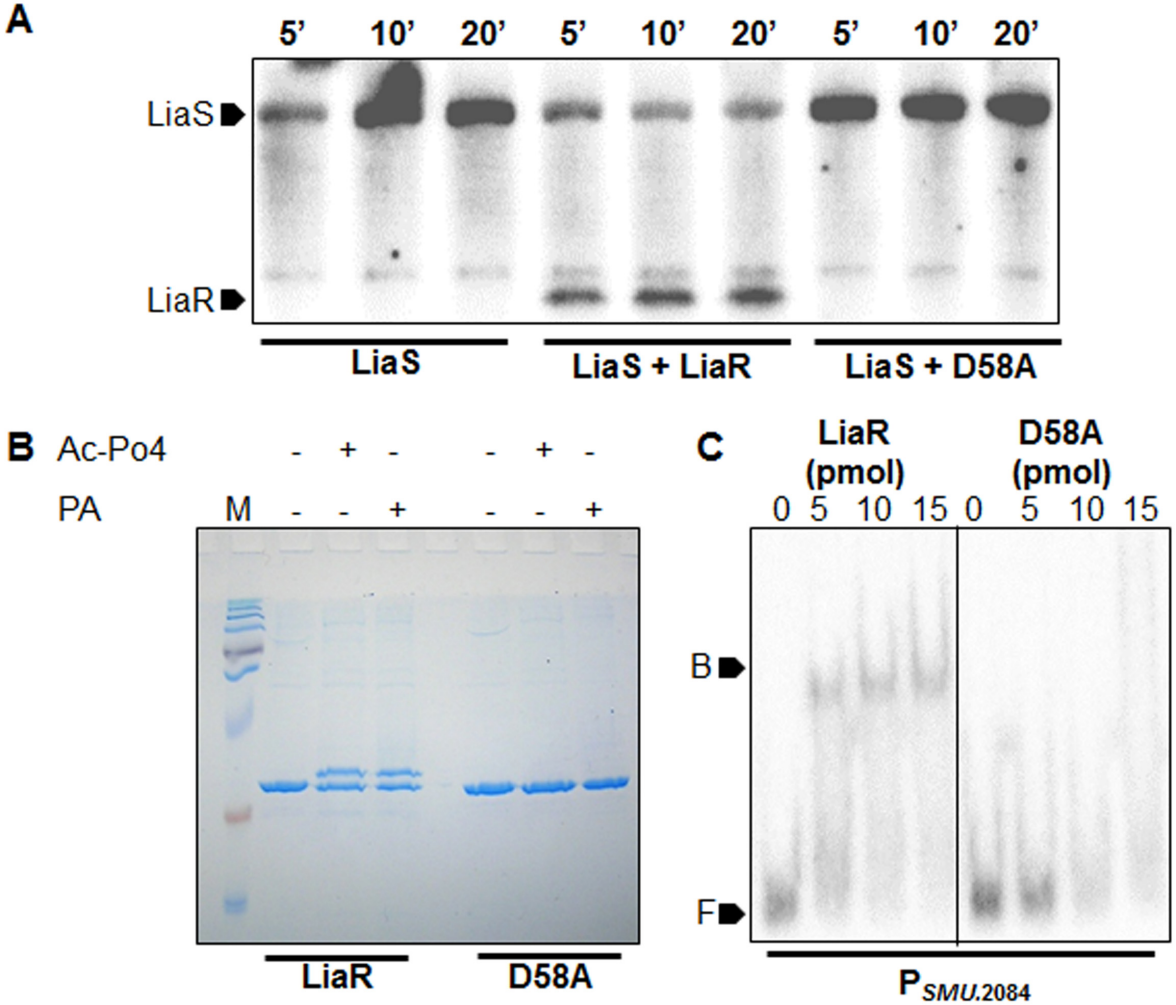
Mutation D58A in LiaR affects phosphorylation and DNA binding ability. (A) Phosphorylated LiaS was incubated with LiaR (1:2 molar ratio) in phosphorylation buffer 1. Samples were drawn at indicated time points and resolved on 10% SDS-PAGE gels. (B) ~5 μg of purified His-LiaR / D58A were incubated in phosphorylation buffer 2 [50 mM Tris-Cl, 50 mM KCl, 20 mM MnCl2, 1 mM DTT at pH 7.5 containing either 50 mM acetyl phosphate (Ac-Po4) or 20 mM phosphoamidate (PA) as phosphodonors] for 1 h at 37°C and then resolved by PhosTag-SDS PAGE. (C) ~0.5 pmol P_*SMU*.2084_ end labelled with γ^32^P-dATP were incubated with ~5, 10 and 15 pmol of purified His-LiaR / His-D58A in binding buffer (20 mM Tris-Cl, 100 mM NaCl, 0.01 mM DTT, 5% glycerol (vol/vol), 1 mM EDTA, 0.01 mg/ml BSA, 5 mM MgCl_2_, and 10 μg/ml Poly(dI-dC) at pH 7.5) for 30 min and then resolved on EMSA gels (5.5% (w/vol) polyacrylamide gels containing 5% glycerol (vol/vol) using 0.5x TBE buffer with 5% glycerol (vol/vol)). Marker F indicates free DNA, while marker B indicates the DNA-protein complex.

A point mutation at position 173 was introduced in the LiaR ORF from A→C resulting in a mutant LiaR having an alanine residue at position 58 of the LiaR protein sequence instead of a conserved aspartic acid residue. We verified if D58A could accept phosphate from the LiaS by phosphotransfer. As inferred from Fig 2A, while the wild-type LiaR could accept the phosphate transferred by LiaS, the D58A mutant was unable to do so. Several studies suggest that response regulators can also be phosphorylated *in-vivo* by accepting phosphate from small molecule phosphodonors [26, 32]. The ability of wild-type LiaR and its D58A mutant to acquire phosphate from small phosphate donors such as acetyl phosphate and phosphoamidate was assessed by Phos-Tag SDS-PAGE [27]. The wild-type protein readily acquired phosphate from both acetyl phosphate and phosphoamidate as indicated by gel retardation during protein electrophoresis in the presence of Phos-Tag [27, 33]. On the other hand, Lia-D58A remained unphosphorylated as revealed by the absence of a mobility shift during Phos-Tag SDS-PAGE (Fig 2B). Since phosphorylation of response regulators plays a key role in DNA binding, we tested the DNA binding abilities of the wild-type and the D58A mutant LiaR proteins. EMSA with an ~200 bp promoter region of SMU.2084 (a previously known direct regulon of LiaR, [5]), indicated that the LiaR-D58A mutant was incapable of binding to the promoter while the wild-type LiaR protein bound well (Fig 2C). These findings highlighted the essentiality of LiaR phosphorylation for DNA binding and direct regulation of the downstream genes. Since the phosphorylation cascade leads to eventual binding of the response regulator to target DNA, we investigated the other genes that are proposed to be under the control of the LiaSR system in *S*. *mutans*. This would allow us to segregate the direct regulons from the indirect regulons and to generate a true LiaR binding sequence.

### LiaR binds the promoters of SMU.753, SMU.1727 and SMU.2084, but is unable to bind the promoter of *lia* (SMU.485)

**Fig 3:**
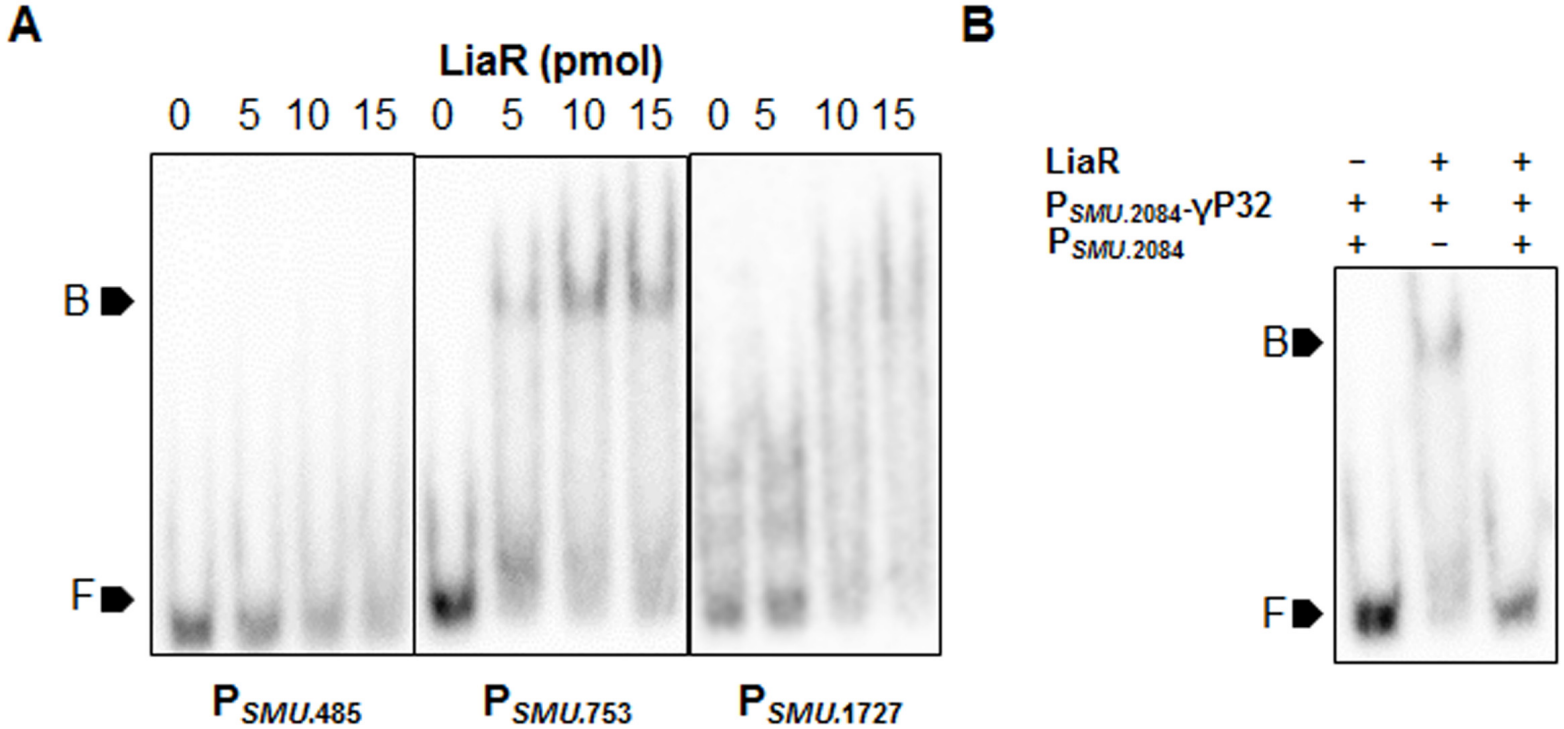
LiaR specifically binds the promoters of *SMU*.753, *SMU*.1727 and *SMU*.2084 but is unable to bind P_*SMU*.*485*_. (A) ~0.5 pmol of P_*SMU*.*485*_, P_*SMU*.753_ and P_*SMU*.1727_ end labelled with γ^32^P-dATP were incubated with ~5, 10 and 15 pmol of purified His-LiaR in binding buffer for 30 min. (B) Addition of non-radiolabelled P_*SMU*.2084_ as cold competitor, two-fold in excess of radiolabelled P_*SMU*.2084_ abolished the gel shift. Both reaction products were resolved on EMSA gels. Marker F indicates free DNA, while marker B indicates the DNA-protein complex.

Some studies in *S*. *mutans* and other organisms have suggested that LiaR regulates its own operon (*liaFSR*: SMU.485, SMU486, SMU.487) [5, 22]. In *S*. *mutans* it was also proposed that LiaSR modulates expression of SMU.2084, SMU.753, SMU.751 and SMU.1727 gene; however, LiaR binding to the promoters of these genes has not been demonstrated. We tested if LiaR could bind to the promoters of these genes by EMSA. We found that LiaR bound the promoters of SMU.753, SMU.2084 and SMU.1727 as suggested earlier (Figs 2C and 3A). Binding was found to be specific, since all EMSA reactions contained poly (dI-dC) as a non-specific competitor. Moreover, addition of excessive non-radiolabelled, but specificprobes abolished binding (Fig 3B). Surprisingly, we found that LiaR was unable to bind P_*lia*_ (Fig 3A) and P_*SMU*.*751*_ (data not shown). Considering the fact that an alternative start codon and promoter have been proposed [5] for the *lia* operon (SMU.485), we also tested a 200bp region upstream of the alternative start codon for LiaR binding. LiaR was also unable to bind the alternative promoter that was proposed earlier as well (data not shown). Furthermore, we also tested another region extending 200bp upstream into the *pknB* ORF located upstream of the *lia* operon for LiaR binding (data not shown). This suggested that LiaR, unlike most TCS may not auto regulate its own expression, at least not directly. This led us to further probe the promoters to which, LiaR binds in order to determine a conserved LiaR-binding motif.

### LiaR specifically binds a 25bp-conserved motif upstream of its target promoters

**Fig 4:**
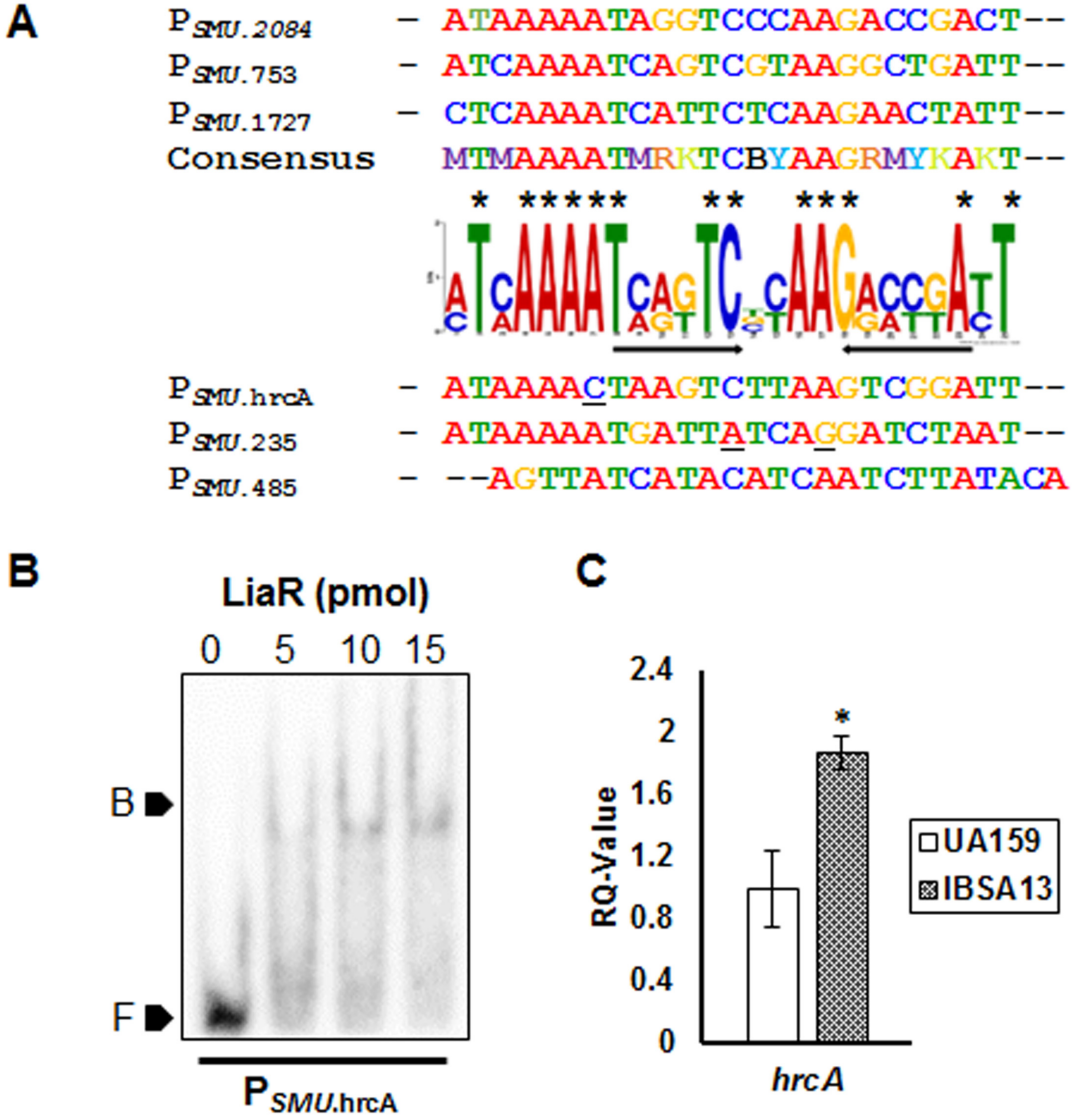
A conserved 25 bp motif is essential for LiaR binding and is present upstream of regulons directly under LiaR control. (A) Predicted LiaR binding motif found using MEME (Multiple Em for Motif Elicitation) in P_*SMU*.2084_, P_*SMU*.753_, and P_*SMU*.1727_. Arrows indicate the position of the inverted repeat while * indicates highly conserved positions. High-scoring motifs found upstream of potential new LiaR regulons *SMU*.235 and *SMU*.*hrcA* are also shown. Bases in the P_*SMU*.hrcA_ and P_*SMU*.235_ motifs that vary from conserved positions are underlined. (B) ~0.5 pmol of P_*SMU*.hrcA_, end labelled with γ^32^P-dATP was incubated with ~5, 10 and 15 pmol of purified His-LiaR in binding buffer for 30 min and then resolved on EMSA gels. Marker F indicates free DNA, while marker B indicates the DNA-protein complex. (C) Quantification of *hrcA* expression in the ΔliaR strain IBSA13 relative to the wildtype strain UA159. Data shown is the mean ± SD of triplicate measurements and *rpoB* was used as an endogenous control. *, significant difference in relation to the wildtype (P<0.05) using a Student’s t-test.

We analyzed the promoter sequences to which LiaR bound by the MEME suite and identified a 25-bp consensus sequence containing a 16-bp inverted-repeat (IR) (Fig 4A). The 25-bp consensus had 13 positions that were fully conserved in all the promoters analyzed. The *S*. *mutans* UA159 genome was analyzed for occurrence of this consensus using FIMO [34]. This search identified two additional promoters, P_*hrcA*_ and P_*SMU*.*235*_, as potential LiaR binding sites. The motif identified in the promoter of *hrcA* had a p-value of 6.53E^-8^ and had 12 out of the 13 conserved residues unchanged. On the other hand, the motif in P_*SMU*.*235*_ had a much lower p-value of 6.85E^-06^ and had only 10 out of the 13 conserved positions unchanged. EMSA with ~200-bp promoter regions of *hrcA* and SMU.235 indicated that LiaR bound to the promoter region of *hrcA* (Fig 4B) while it was unable to bind to the promoter of SMU.235 (data not shown). To confirm if *hrcA* was indeed a direct regulon of LiaR, we also performed quantitative PCR to determine the expression level of *hrcA* in a LiaR-deficient strain (IBSA13) relative to the wild type parent. The expression of *hrcA* was elevated in IBSA13 as compared to UA159 indicating that LiaR likely acts as a repressor of *hrcA* in *S*. *mutans* (Fig 4C).

**Fig 5:**
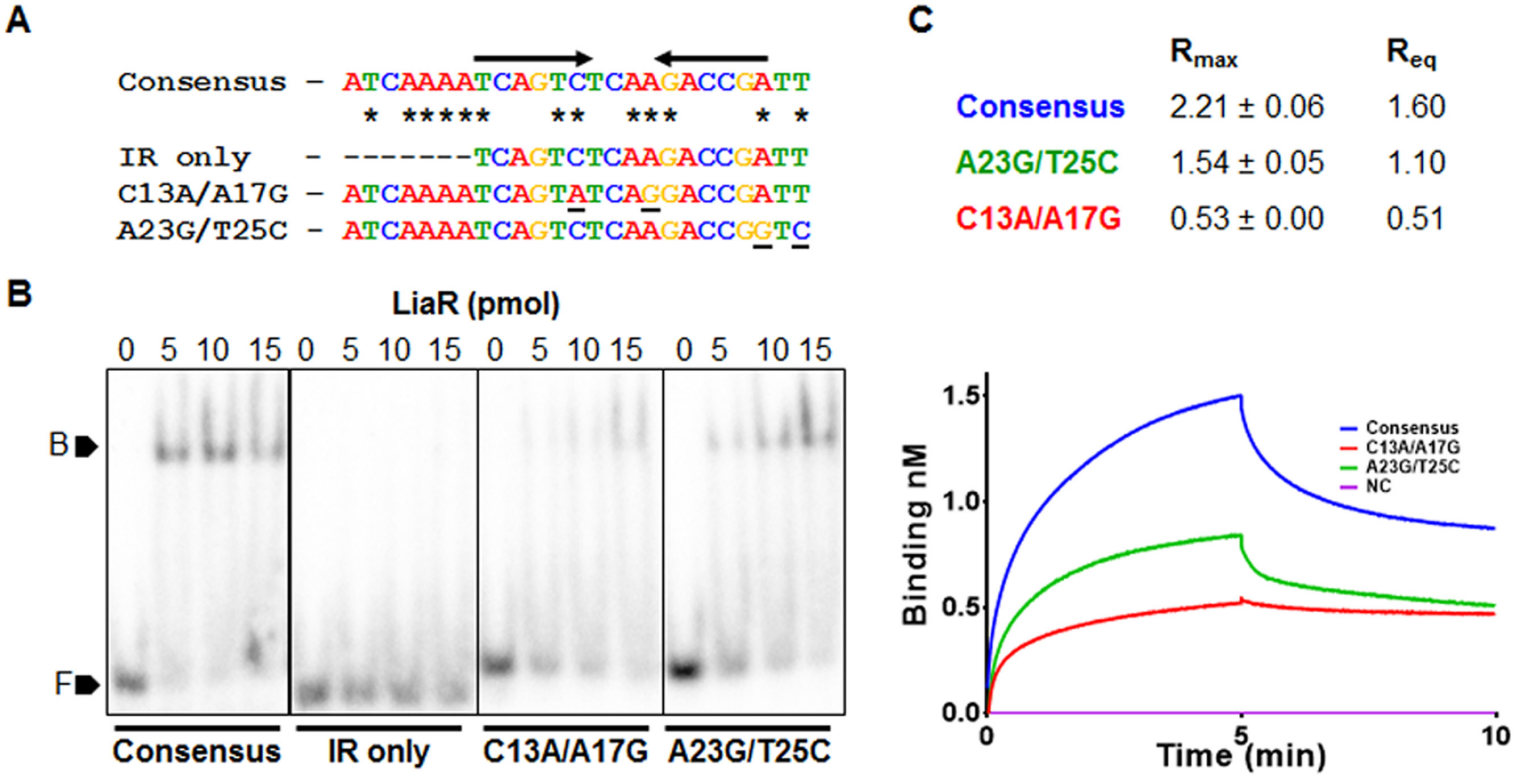
Alterations in conserved residues of the predicted LiaR binding motif affect binding by LiaR variably (A) The LiaR binding consensus identified in this study was altered by introducing substitutions at fully conserved postions 13,17, 23 and 25. Additionally the binding ability of the inverted repeat alone to LiaR was assessed. (B) ~1 pmol of indicated, annealed oligos (Consensus, IR only, C13A/A17G, and A23G/T25C), end labelled with γ^32^P-dATP were incubated with ~15, 20 and 30 pmol of purified His-LiaR in binding buffer for 30 min and then resolved on 7.5% EMSA gels. Marker F indicates free DNA, while marker B indicates the DNA-protein complex. (C) Biotinylated consensus, C13A/A17G and A23G/T25C oligos were immobilized on streptavidin biosensors and then exposed to 1 μM LiaR, prepared in binding buffer for a period of 5 minutes to allow association followed by a 5 min exposure to binding buffer to allow complex dissociation. The maximum binding ability (Rmax and the equilibrium binding ability (Req) were calculated automatically and reported.

The fact that LiaR was unable to bind P_*SMU*.*235*_ led us to question the essentiality of various positions in the LiaR-binding motif (synthetic consensus generated from LiaR-binding promoters) for effective binding to LiaR. We noted that the motif identified in P_*SMU*.*235*_ had C13A and A17G substitutions. In addition to these alterations, we introduced changes in the 25-bp pattern at fully conserved positions (A23G and T25C) and assessed the binding of the modified consensus sequences to LiaR (Fig 5A). LiaR was found to bind the original consensus most effectively at the lowest tested protein concentration (15pmols) while it bound to the consensus with the C13A/A17G alteration at much lower a level only at twice the protein concentration. These binding studies correlated well with our earlier observation since this substitution occurred naturally in P_*SMU*.*235*_ to which LiaR did not bind. LiaR binding to the A23G/T25C altered consensus was better than the C13A/A17G altered consensus but lower than the original sequence (Fig 5B). To confirm the observation, we measured the R_max_ and R_eq_ of the altered LiaR binding motifs by biolayer interferometry (BLI). We used biotinylated DNA fragments as the ligands, bound to streptavidin biosensors and the LiaR protein as the analyte in solution. We found that the R_max_ and R_eq_ values of the C13A/A17G consensus were reduced by 4- and 3-fold, respectively, relative to the original motif. This observation was in agreement with our EMSA studies. Similarly, the R_max_ and R_eq_ values for the A23G/T25C motif were reduced ~1.5 fold, relative to the original motif (Fig 5C). Since an earlier study on lactococci proposed a 16-bp IR as the putative LiaR binding site, we wanted to test whether LiaR could bind to the IR that we detected [5]. When we performed EMSA with just the IR consensus sequence, we found that LiaR could not bind to the 16-bp IR alone suggesting that the entire 25 bp sequence is essential for LiaR binding (Fig 5B).

## Discussion

The LiaSR TCS controls the response to cell-wall stress *via* direct or indirect regulatory networks in several *Firmicutes*. This system is induced upon exposure of the cell to bacitracin like antibiotics that target the lipid II cycle in cell wall biogenesis [35]. Several studies have been conducted in *S*. *mutans* and in other bacteria to determine the regulons under the control of the LiaSR TCS. The LiaR regulon in *S*. *mutans* and *S*. *pneumoniae* have been characterized earlier [6, 22]. About 174 genes (~5% of the genome) including genes contributing to stress resistance and DNA uptake were altered in expression in *S*. *mutans* upon LiaR deletion, while several genes involved in stress tolerance were found to be altered in expression in *S*. *pneumoniae* [6, 22]. Based on a consensus derived from *B*. *subtilis* LiaR regulons, identified by microarray analyses Jordon and colleagues have predicted a 28-bp long *B*. *subtilis* LiaR-binding motif [9, 11]. This strategy was later expanded to identify LiaR binding motifs in lactococci and streptococci [5, 6]. However, occurrence of these motifs on the genome of these bacteria were limited to the promoters of a few genes suggesting that most of the LiaR regulons identified in microarray studies could be regulated indirectly. In addition, the reported motifs upstream of newly identified LiaR regulons in *S*. *mutans* were not well conserved. Notably, the motif reported to be upstream of the autoregulated *liaFSR* operon was altered at two out of six key positions that were fully conserved in all other motifs [5]. The LiaR regulon also includes other TCS and a few transcriptional regulators, which may up- or down-regulate their target regulons leading to a much-enhanced effect upon LiaR inactivation [22]. Considering the existing ambiguities in the LiaR-binding motif, we revisited the functioning of the LiaSR system in an attempt to segregate the direct regulons of LiaR and determine the actual LiaR binding motif in *S*. *mutans*.

Much like other known TCS, we found that LiaS can autophosphorylate in the presence of ATP and then transfer the phosphate group to LiaR. The phosphorylation of LiaR is critical, since phophorylation of *B*. *subtilis* LiaR has been shown to be essential for dimerization and binding to the target promoters [19]. While response regulators acquire phosphate group from their cognate sensor kinases, it is also possible for the response regulators to be phosphorylated in the presence of cellular small molecule phosphate donors such as acetyl phosphate [36]. Our results showed that *S*. *mutans* LiaR readily acquired phosphate from both acetyl phosphate and phosphoamidate. Response regulators can also be phosphoryaled by non-cognate sensor kinases by a process known as cross-talk [37]. Our earlier studies show that inactivation of *liaS* or *liaR* in *S*. *mutans* leads to different effects on virulence gene expression suggesting cross-talk between LiaSR and other TCS [21]. Usually the cross-talk influenced by other TCS and cellular acetyl phosphate is avoided by the additional phosphatase activity exhibited by the cognate sensor kinase. *B*. *subtilis* LiaS has been shown to have phosphatase activity in the absence of environmental stimulus and effectively dephosphorylates LiaR that might have been phosphorylated by cross-talk mechanism [19]. Based on our phosphotransfer assay, we believe that *S*. *mutans* LiaS does not display a robust phosphatase activity (Fig 1). Phosphorylated LiaS is very stable (at least for an hour) in the absence of cognate LiaR. We also found that the phosphotransfer reaction was relatively fast and that over 50% of the transfer occurs within 5 min (Fig 1). Since phosphorylated LiaS failed to transfer the phosphate group to CovR, the reaction seems to be very specific (data not shown). It also appears that phosphorylated LiaR is relatively stable and the presence of LiaS did not dephosphorylate LiaR.

Altering the conserved aspartic acid (the site of phosphorylation) usually results in inability to acquire phosphate group thereby interfering with the dimerization and DNA binding. The D58A mutant of LiaR was found to be incapable of acquiring phosphate from both its cognate LiaS and the small molecule phosphodonors tested. Mutation D58A also rendered the protein unable to bind its target promoters indicating that phosphorylation is required for dimerization and DNA binding. It is to be noted that in *S*. *mutans*, LiaSR are expressed from the *liaFSR* operon. LiaF, the third component of this system, has been shown to play a role in the functioning of the LiaSR TCS in *B*. *subtilis*, *L*. *monocytogenes*, and in some streptococci, where it acts as a negative regulator of the *liaFSR* operon [6, 9, 17]. Earlier reports have predicted the occurrence of LiaR-binding motifs in a genomic context in *S*. *mutans*, *S*. *pneumoniae*, *B*. *subtilis* and lactococci where a LiaR-binding motif in the promoter of the *liaFSR* operon has been predicted. Quite contrary to these predictions, our DNA binding studies indicated that *S*. *mutans* LiaR could not bind the promoter of *liaFSR*. Though in-frame deletion of *liaR* in streptococci has been shown to cause perturbations in the expression levels of *liaFSR*, our data suggest that the regulation of *liaFSR* by LiaR may not be direct as the promoter of *liaFSR* lacks a LiaR-binding motif.

Three direct regulons of LiaR, verified by DNA-binding studies SMU.2084, SMU.753 and SMU.1727 were found to encode SpxB, PspC protein and an OxaA class protein, respectively. All of these proteins have been implicated in stress tolerance in independent studies. *spxB* in *S*. *mutans* was shown to play a minor role in oxidative stress tolerance and played a key role in maintaining cell wall homeostasis [38]. Similarly, PspC homologs have shown to be induced in the presence of ethanol, heat and osmotic shock; and has been proposed as a vaccine candidate in pneumococci [39]. OxaA class proteins on the other hand have been implicated in membrane protein biogenesis, tolerance to acid stress and are known to bind *E*. *coli* ribosomes [40]. In this study we identified another direct LiaR regulon, *hrcA*, which encodes a regulatory protein that coordinates response to thermal stress. Lack of HrcA is known to cause increased sensitivity to acid stress due to lower chaperone and ATPase activity [41]. It has been proposed that this effect is a result of the *groEL* and *dnaK* chaperone encoding operons being regulated by HrcA [42]. Our findings along with earlier data suggest that LiaR globally administers response to a variety of stresses encountered by *S*. *mutans* in its environment by direct regulation of some key transcriptional regulators that modulate stress responses. All the direct regulons of LiaR verified by DNA binding contained the newly defined 25 bp LiaR-binding motif identified in this study. By a combination of introducing mutations and deleting portions of this newly defined conserved LiaR-binding motif, we showed that mutations within the 16bp inverted repeat have a negative effect on LiaR binding. Removal of the conserved sequence “MTMA_4_” upstream of the inverted repeat resulted in LiaR being unable to bind to the motif. The fact that the *B*.*subtilis* LiaR-binding motif also contains a stretch of adenines next to the inverted repeat is noteworthy here [9, 11]. The newly defined LiaR-binding motif is most similar to the CesR binding motif (TCAGHCTnnAGDCTGA) reported in lactococci except for the MTMA_4_ stretch. However, closer inspection of documented CesR-binding promoters indicated that the inverted repeat projected as the CesR-binding motif contained an A_4_ stretch on one of the ends [43]. Thus LiaR binding to target promoters depends on the occurrence of the entire 25bp conserved motif including the 16-bp inverted repeats and the binding affinities might vary across the regulons as a result of minor alterations in the binding sequences.

## References

[1] Boll JM, Hendrixson DR. A specificity determinant for phosphorylation in a response regulator prevents in vivo cross-talk and modification by acetyl phosphate. Proceedings of the National Academy of Sciences of the United States of America. 2011;108(50):20160–5. 10.1073/pnas.1113013108 22128335 PMC3250149

[2] Beier D, Gross R. Regulation of bacterial virulence by two-component systems. Current opinion in microbiology. 2006;9(2):143–52. 10.1016/j.mib.2006.01.005 . 16481212

[3] Gotoh Y, Eguchi Y, Watanabe T, Okamoto S, Doi A, Utsumi R. Two-component signal transduction as potential drug targets in pathogenic bacteria. Current opinion in microbiology. 2010;13(2):232–9. 10.1016/j.mib.2010.01.008 . 20138000

[4] Worthington RJ, Blackledge MS, Melander C. Small-molecule inhibition of bacterial two-component systems to combat antibiotic resistance and virulence. Future medicinal chemistry. 2013;5(11):1265–84. 10.4155/fmc.13.58 . 23859207

[5] Suntharalingam P, Senadheera MD, Mair RW, Levesque CM, Cvitkovitch DG. The LiaFSR system regulates the cell envelope stress response in Streptococcus mutans. Journal of bacteriology. 2009;191(9):2973–84. 10.1128/JB.01563-08 19251860 PMC2681809

[6] Eldholm V, Gutt B, Johnsborg O, Bruckner R, Maurer P, Hakenbeck R, et al The pneumococcal cell envelope stress-sensing system LiaFSR is activated by murein hydrolases and lipid II-interacting antibiotics. Journal of bacteriology. 2010;192(7):1761–73. 10.1128/JB.01489-09 20118250 PMC2838051

[7] Yin S, Daum RS, Boyle-Vavra S. VraSR two-component regulatory system and its role in induction of pbp2 and vraSR expression by cell wall antimicrobials in Staphylococcus aureus. Antimicrobial agents and chemotherapy. 2006;50(1):336–43. 10.1128/AAC.50.1.336-343.2006 16377706 PMC1346790

[8] Kuroda M, Kuroda H, Oshima T, Takeuchi F, Mori H, Hiramatsu K. Two-component system VraSR positively modulates the regulation of cell-wall biosynthesis pathway in Staphylococcus aureus. Molecular microbiology. 2003;49(3):807–21. . 12864861 10.1046/j.1365-2958.2003.03599.x

[9] Jordan S, Junker A, Helmann JD, Mascher T. Regulation of LiaRS-dependent gene expression in bacillus subtilis: identification of inhibitor proteins, regulator binding sites, and target genes of a conserved cell envelope stress-sensing two-component system. Journal of bacteriology. 2006;188(14):5153–66. 10.1128/JB.00310-06 16816187 PMC1539951

[10] Mascher T, Zimmer SL, Smith TA, Helmann JD. Antibiotic-inducible promoter regulated by the cell envelope stress-sensing two-component system LiaRS of Bacillus subtilis. Antimicrobial agents and chemotherapy. 2004;48(8):2888–96. 10.1128/AAC.48.8.2888-2896.2004 15273097 PMC478541

[11] Jordan S, Rietkotter E, Strauch MA, Kalamorz F, Butcher BG, Helmann JD, et al LiaRS-dependent gene expression is embedded in transition state regulation in Bacillus subtilis. Microbiology. 2007;153(Pt 8):2530–40. . 17660417 10.1099/mic.0.2007/006817-0

[12] Butcher BG, Lin YP, Helmann JD. The yydFGHIJ operon of Bacillus subtilis encodes a peptide that induces the LiaRS two-component system. Journal of bacteriology. 2007;189(23):8616–25. 10.1128/JB.01181-07 17921301 PMC2168931

[13] Klinzing DC, Ishmael N, Dunning Hotopp JC, Tettelin H, Shields KR, Madoff LC, et al The two-component response regulator LiaR regulates cell wall stress responses, pili expression and virulence in group B Streptococcus. Microbiology. 2013;159(Pt 7):1521–34. 10.1099/mic.0.064444-0 23704792 PMC3749725

[14] McCallum N, Meier PS, Heusser R, Berger-Bachi B. Mutational analyses of open reading frames within the vraSR operon and their roles in the cell wall stress response of Staphylococcus aureus. Antimicrobial agents and chemotherapy. 2011;55(4):1391–402. 10.1128/AAC.01213-10 21220524 PMC3067146

[15] Galbusera E, Renzoni A, Andrey DO, Monod A, Barras C, Tortora P, et al Site-specific mutation of Staphylococcus aureus VraS reveals a crucial role for the VraR-VraS sensor in the emergence of glycopeptide resistance. Antimicrobial agents and chemotherapy. 2011;55(3):1008–20. 10.1128/AAC.00720-10 21173175 PMC3067069

[16] Qureshi NK, Yin S, Boyle-Vavra S. The role of the Staphylococcal VraTSR regulatory system on vancomycin resistance and vanA operon expression in vancomycin-resistant Staphylococcus aureus. PloS one. 2014;9(1):e85873 10.1371/journal.pone.0085873 24454941 PMC3893269

[17] Fritsch F, Mauder N, Williams T, Weiser J, Oberle M, Beier D. The cell envelope stress response mediated by the LiaFSRLm three-component system of Listeria monocytogenes is controlled via the phosphatase activity of the bifunctional histidine kinase LiaSLm. Microbiology. 2011;157(Pt 2):373–86. 10.1099/mic.0.044776-0 . 21030435

[18] Munita JM, Mishra NN, Alvarez D, Tran TT, Diaz L, Panesso D, et al Failure of high-dose daptomycin for bacteremia caused by daptomycin-susceptible Enterococcus faecium harboring LiaSR substitutions. Clinical infectious diseases: an official publication of the Infectious Diseases Society of America. 2014;59(9):1277–80. 10.1093/cid/ciu642 25107294 PMC4271039

[19] Schrecke K, Jordan S, Mascher T. Stoichiometry and perturbation studies of the LiaFSR system of Bacillus subtilis. Molecular microbiology. 2013;87(4):769–88. 10.1111/mmi.12130 . 23279150

[20] Zhang J, Biswas I. A phenotypic microarray analysis of a Streptococcus mutans liaS mutant. Microbiology. 2009;155(Pt 1):61–8. Epub 2009/01/02. 155/1/61 [pii] 10.1099/mic.0.023077-0 . 19118347 PMC2814309

[21] Chong P, Drake L, Biswas I. LiaS regulates virulence factor expression in Streptococcus mutans. Infection and immunity. 2008;76(7):3093–9. 10.1128/IAI.01627-07 18458070 PMC2446727

[22] Perry JA, Levesque CM, Suntharaligam P, Mair RW, Bu M, Cline RT, et al Involvement of Streptococcus mutans regulator RR11 in oxidative stress response during biofilm growth and in the development of genetic competence. Lett Appl Microbiol. 2008;47(5):439–44. Epub 2009/01/17. LAM2455 [pii] 10.1111/j.1472-765X.2008.02455.x . 19146535 PMC2771662

[23] Banerjee A, Biswas I. Markerless multiple-gene-deletion system for Streptococcus mutans. Applied and environmental microbiology. 2008;74(7):2037–42. Epub 2008/02/12. AEM.02346-07 [pii] 10.1128/AEM.02346-07 . 18263742 PMC2292598

[24] Belcheva A, Golemi-Kotra D. A close-up view of the VraSR two-component system. A mediator of Staphylococcus aureus response to cell wall damage. The Journal of biological chemistry. 2008;283(18):12354–64. 10.1074/jbc.M710010200 . 18326495

[25] Silversmith RE, Appleby JL, Bourret RB. Catalytic mechanism of phosphorylation and dephosphorylation of CheY: kinetic characterization of imidazole phosphates as phosphodonors and the role of acid catalysis. Biochemistry. 1997;36(48):14965–74. . 9398221 10.1021/bi9715573

[26] Lukat GS, McCleary WR, Stock AM, Stock JB. Phosphorylation of bacterial response regulator proteins by low molecular weight phospho-donors. Proceedings of the National Academy of Sciences of the United States of America. 1992;89(2):718–22. 1731345 10.1073/pnas.89.2.718 PMC48310

[27] Barbieri CM, Stock AM. Universally applicable methods for monitoring response regulator aspartate phosphorylation both in vitro and in vivo using Phos-tag-based reagents. Analytical biochemistry. 2008;376(1):73–82. Epub 2008/03/11. S0003-2697(08)00073-0 [pii] 10.1016/j.ab.2008.02.004 . 18328252 PMC2504525

[28] Biswas S, Biswas I. Regulation of the glucosyltransferase (gtfBC) operon by CovR in Streptococcus mutans. Journal of bacteriology. 2006;188(3):988–98. . 16428403 10.1128/JB.188.3.988-998.2006 PMC1347363

[29] Bailey TL, Boden M, Buske FA, Frith M, Grant CE, Clementi L, et al MEME SUITE: tools for motif discovery and searching. Nucleic acids research. 2009;37(Web Server issue):W202–8. 10.1093/nar/gkp335 19458158 PMC2703892

[30] Concepcion J, Witte K, Wartchow C, Choo S, Yao D, Persson H, et al Label-free detection of biomolecular interactions using BioLayer interferometry for kinetic characterization. Combinatorial chemistry & high throughput screening. 2009;12(8):791–800. . 19758119 10.2174/138620709789104915

[31] Dmitriev A, Mohapatra SS, Chong P, Neely M, Biswas S, Biswas I. CovR-Controlled Global Regulation of Gene Expression in Streptococcus mutans. PloS one. 2011;6(5):e20127 Epub 2011/06/10. 10.1371/journal.pone.0020127PONE-D-11-03251 [pii]. . 21655290 PMC3105014

[32] Scharf BE. Summary of useful methods for two-component system research. Current opinion in microbiology. 2010;13(2):246–52. 10.1016/j.mib.2010.01.006 . 20138001

[33] Kinoshita E, Kinoshita-Kikuta E, Koike T. Phos-tag SDS-PAGE systems for phosphorylation profiling of proteins with a wide range of molecular masses under neutral pH conditions. Proteomics. 2012;12(2):192–202. 10.1002/pmic.201100524 . 22121028

[34] Grant CE, Bailey TL, Noble WS. FIMO: scanning for occurrences of a given motif. Bioinformatics. 2011;27(7):1017–8.: 10.1093/bioinformatics/btr064 21330290 PMC3065696

[35] Mascher T, Margulis NG, Wang T, Ye RW, Helmann JD. Cell wall stress responses in Bacillus subtilis: the regulatory network of the bacitracin stimulon. Molecular microbiology. 2003;50(5):1591–604. . 14651641 10.1046/j.1365-2958.2003.03786.x

[36] Gao R, Stock AM. Biological insights from structures of two-component proteins. Annual review of microbiology. 2009;63:133–54. Epub 2009/07/07. 10.1146/annurev.micro.091208.073214 . 19575571 PMC3645274

[37] Laub MT, Goulian M. Specificity in two-component signal transduction pathways. Annu Rev Genet. 2007;41:121–45. Epub 2007/12/14. 10.1146/annurev.genet.41.042007.170548 . 18076326

[38] Kajfasz JK, Rivera-Ramos I, Abranches J, Martinez AR, Rosalen PL, Derr AM, et al Two Spx proteins modulate stress tolerance, survival, and virulence in Streptococcus mutans. Journal of bacteriology. 2010;192(10):2546–56. 10.1128/JB.00028-10 20233935 PMC2863552

[39] Schachern PA, Tsuprun V, Ferrieri P, Briles DE, Goetz S, Cureoglu S, et al Pneumococcal PspA and PspC proteins: potential vaccine candidates for experimental otitis media. International journal of pediatric otorhinolaryngology. 2014;78(9):1517–21. 10.1016/j.ijporl.2014.06.024 25015773 PMC4129636

[40] Wu ZC, de Keyzer J, Berrelkamp-Lahpor GA, Driessen AJ. Interaction of Streptococcus mutans YidC1 and YidC2 with translating and nontranslating ribosomes. Journal of bacteriology. 2013;195(19):4545–51. 10.1128/JB.00792-13 23935050 PMC3807456

[41] Schulz A, Schumann W. hrcA, the first gene of the Bacillus subtilis dnaK operon encodes a negative regulator of class I heat shock genes. Journal of bacteriology. 1996;178(4):1088–93. Epub 1996/02/01. . 8576042 10.1128/jb.178.4.1088-1093.1996 PMC177769

[42] Lemos JA, Chen YY, Burne RA. Genetic and physiologic analysis of the groE operon and role of the HrcA repressor in stress gene regulation and acid tolerance in Streptococcus mutans. Journal of bacteriology. 2001;183(20):6074–84. . 11567008 10.1128/JB.183.20.6074-6084.2001 PMC99687

[43] Roces C, Campelo AB, Veiga P, Pinto JP, Rodriguez A, Martinez B. Contribution of the CesR-regulated genes llmg0169 and llmg2164-2163 to Lactococcus lactis fitness. International journal of food microbiology. 2009;133(3):279–85. 10.1016/j.ijfoodmicro.2009.06.002 . 19559493

